# Heterologous Expression of the Thiopeptide Antibiotic GE2270 from *Planobispora rosea* ATCC 53733 in *Streptomyces coelicolor* Requires Deletion of Ribosomal Genes from the Expression Construct

**DOI:** 10.1371/journal.pone.0090499

**Published:** 2014-03-05

**Authors:** Katrin Flinspach, Claudia Kapitzke, Arianna Tocchetti, Margherita Sosio, Alexander K. Apel

**Affiliations:** 1 Pharmaceutical Institute, University of Tübingen, Tübingen, Germany; 2 German Centre for Infection Research (DZIF), partner site Tübingen, Tübingen, Germany; 3 KtedoGen Srl, Milan, Italy; 4 NAICONS Srl, Milan, Italy; University Paris South, France

## Abstract

GE2270 is a thiopeptide antibiotic generated by extensive posttranslational modifications of a ribosomally generated precursor peptide. Thiopeptides are especially active against Gram-positive bacteria, including methicillin resistant *Staphylococcus aureus* (MRSA). In this study the GE2270 biosynthetic gene cluster (*pbt*) from *Planobispora rosea* ATCC 53733 was successfully expressed in the heterologous host strain *Streptomyces coelicolor* M1146. Notably, exconjugants containing the *pbt* gene cluster could only be obtained after deletion of the major part of the ribosomal genes flanking the gene cluster. This is a striking example that genes belonging to primary metabolism can prevent the successful conjugative transfer of DNA from phylogenetic distant species and thus complicate heterologous expression of secondary metabolite gene clusters. GE2270 production in the heterologous producer strain increased after introduction of the constitutive ermE* promoter upstream of the GE2270 resistance gene *tuf* from *P. rosea*. Insertion of the inducible *tcp830* promoter resulted in inducible GE2270 production. When the regulatory gene *pbtR* was deleted, the resulting strain ceased to produce GE2270, suggesting an essential role of PbtR as a putative transcriptional activator of GE2270 expression.

## Introduction

Thiopeptide antibiotics comprise about one hundred natural compounds, including the first representative of the class, micrococcin, discovered in 1948 [1], as well as thiostrepton, thiomuracin or berninamycin. They derive from ribosomally generated precursor peptides with a length of approximately 50–60 amino acids, which are shortened to the 14–18 amino acids C-terminal region forming the final antibiotic after extensive posttranslational modifications [2]–[4].

Thiopeptides are especially active against Gram-positive bacteria, including MRSA, but have also been shown to be active against malaria parasites [5], [6] and to have antiproliferative activity against human cancer cells [7], [8]. They exhibit their antibiotic activity by one of two mechanisms, both inhibiting bacterial protein biosynthesis. One subclass of thiopeptides targets the bacterial elongation factor Tu (EF-Tu), preventing its delivery of aminoacyl-tRNAs to the ribosome. This subclass includes, among others, GE2270, thiomuracin and GE37468 [9]. The other subclass targets the 50S subunit of the ribosome and affects the loops defined by 23S rRNA and the L11 protein. Prominent members of this class are for instance thiostrepton and the thiocillins [10].

Their interesting biological activities make thiopeptides promising drug candidates. The low water solubility and the poor pharmacokinetics of thiopeptides have so far prevented their clinical use, but derivatives of the thiopeptide antibiotic GE2270, produced by the rare actinomycete *Planobispora rosea* ATCC 53733, have now entered clinical testing for the topical treatment of acne [11] or *Clostridium difficile* infections [12]. Structural modification of the GE2270 molecule by genetic engineering may provide a strategy to generate additional compounds of this class for medical use. This has prompted recent efforts directed at the heterologous expression and genetic modification of thiazolylpeptides [13]–[15].

**Figure 1:**
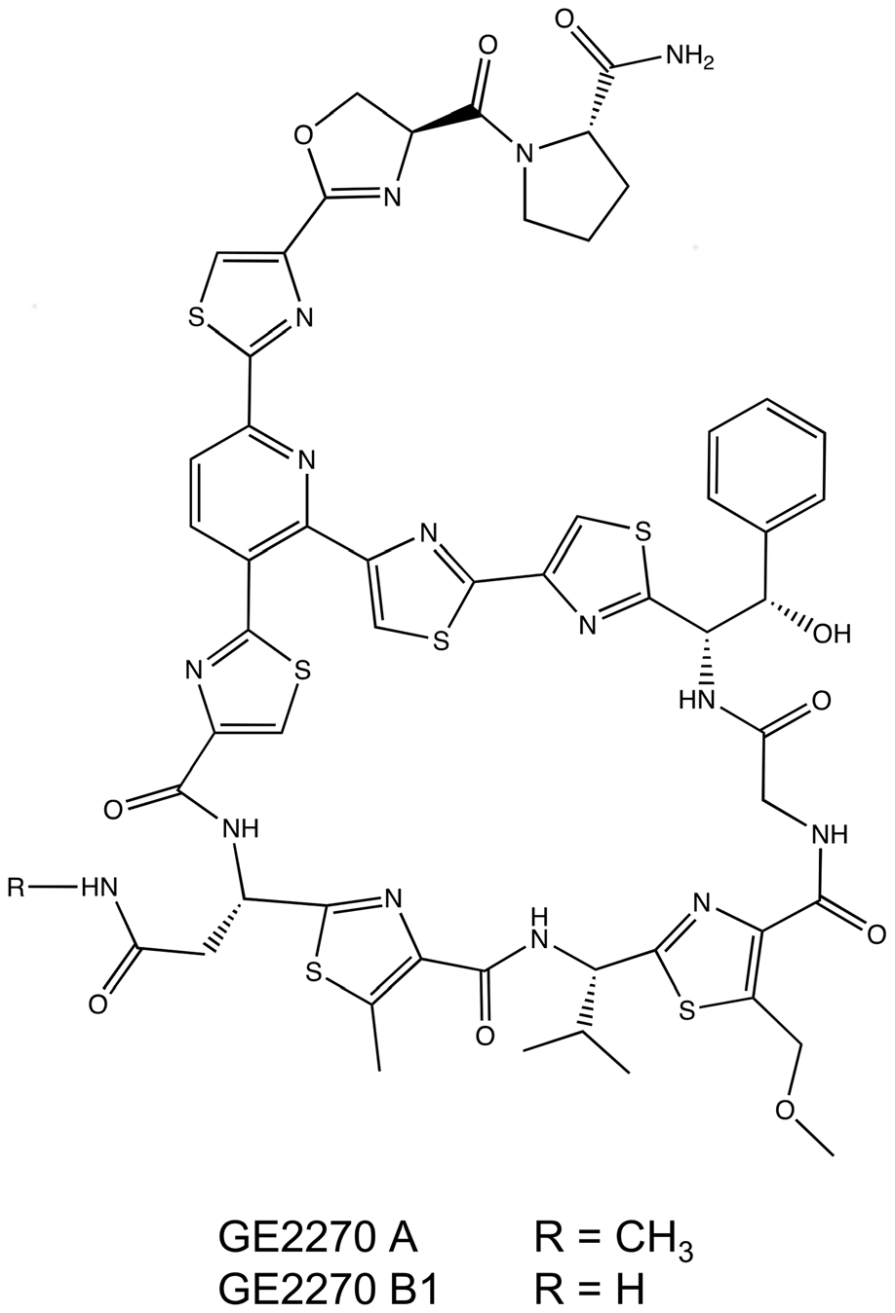
Structures of the two GE2270 congeners described in this study.

GE2270 is naturally produced by *Planobispora rosea* in the form of 10 closely related congeners [16], which differ mainly in their methylation state. The two of them discussed in this study are shown in Figure 1. The main compound produced is GE2270A, which has the highest methylation state and antibacterial activity. All other congeners also exhibit activity against Gram-positive bacteria and their minimum inhibitory concentrations (MIC) are only slightly lower than that of GE2270A [16], [17].

Recently, the biosynthetic gene cluster of GE2270 (*pbt*) in *P. rosea* was discovered and heterologously expressed in *Nonomuraea sp.* ATCC 39727, but attempts on heterologous expression in Streptomycetes, namely *Streptomyces lividans* and *Streptomyces albus*, remained unsuccessful [15]. This cluster shows high sequence identity (between 64–92% on amino acid level) to the GE2270 biosynthetic gene cluster (*tpd*) previously described from *Nonomuraea sp.* WU8817 [18]. It comprises 17 genes, *pbtR*, *pbtG1*, *pbtB1*, *pbtO*, *pbtX*, *pbtM1*, *pbtM2*, *pbtA*-*H*, *pbtM3* and *pbtM4*, all transcribed in the same direction. The putative resistance gene *tuf* (hereafter referred to as *tuf^R^*), coding for a resistant form of EF-Tu, is found in short distance downstream of the cluster.

The gene cluster for another thiopeptide targeting EF-Tu, GE37468 from *Streptomyces* sp. ATCC 55365, has been expressed in *Streptomyces lividans*, as reported by the group of C. T. Walsh in 2011 [13]. However the yield (2–3 mg l^−1^) was lower than in the wild type strain and much lower than GE2270 production in *Nonomuraea* sp. ATCC 39727 (250 mg l^−1^) [15].

Our laboratory successfully employed *Streptomyces coelicolor* host strains (M512, M1146, M1152, M1154) [19], for the heterologous expression of several antibiotic biosynthetic gene clusters, e.g. of aminocoumarins and caprazamycin [21], [22]. Particularly *S. coelicolor* M1146, derived from *S. coelicolor* M145 but lacking the gene clusters for actinorhodin (*act*), undecylprodigiosin (*red*), calcium dependent antibiotic (*cda*) and coelimycin P1 (*cpk*) [19] proved to be a valuable expression host due to its good growth and sporulation and the lower background production of other secondary metabolites. In some cases production of the heterologously expressed antibiotic was achieved or increased by additional use of constitutive [23] or inducible promoters [24].

Especially the successful heterologous production of GE2270 in *Nonomuraea* sp. ATCC 39727 provokes the question why heterologous expression of the *pbt* gene cluster failed in *Streptomyces* strains. Thus, we addressed this question employing the engineered expression host *S. coelicolor* M1146 and the inducible *tcp830* promoter [25]. Heterologous expression of GE2270 in this strain would open a convenient route to the generation of new GE2270 derivatives by genetic methods, since genetic modification and handling is easier for this strain than for *Nonomuraea* sp. ATCC 39727.

## Materials and Methods

### Bacterial strains, cosmids, plasmids and general methods

Bacterial strains, cosmids and plasmids used in this study are listed in Table S1.

*Escherichia coli* strains were cultivated in LB medium [26], *Streptomyces coelicolor* strains were maintained and grown on MS medium [27]. Standard procedures for DNA isolation and manipulation were carried out as described by Sambrook and Russell for *E. coli* [26] and Kieser *et al.* for Actinomycetes [27].

### Production, extraction and detection of GE2270 congeners

Homogenized frozen inoculum was prepared as described before [22], [28]. For standard cultivation each well of 24-square deepwell plates was filled with 3 ml of production medium, as described by Siebenberg *et al.* [28] and inoculated with 7.5 µl of homogenized, frozen inoculum. Cultivation was carried out at 30°C and 300 rpm. Cultivations in 300 ml baffled Erlenmeyer flasks were carried out in 50 ml medium at 30°C and 200 rpm.

Six typical thiopeptide production media (namely “main production medium” described by Selva *et al.* [29], RARE3 [30], Fermentation medium C [31], M8 [32], GE37468 production medium [13] and AF medium [33]), four production media proven for production of secondary metabolites (namely chemically defined medium (CDM) described by Kominek [34], liquid MS medium [27], GYM medium [35] and phenazine production medium [36]) and further 31 production media according to a systematically selected screen media list kindly provided by EntreChem S. L. (Oviedo, Spain) were prepared and tested for GE2270 production. For GE2270 detection 2 ml of culture were pelleted and extracted with 650 µl of methanol by shaking at 1400 rpm and 40°C for 1 hour. 80 µl of the centrifuged supernatant were analyzed by HPLC on an Agilent 1100 system equipped with a reverse phase column (Reprosil-Pur C18 AQ (5 µm), 250 mm×4.6 mm) at a flow rate of 1 ml min^−1^ with a linear gradient from 10% to 90% acetonitrile and 3.16 g l^−1^ ammonium formate in H_2_O over 30 min and UV detection at 310 nm. HPLC-MS analysis was carried out as described before [15].

### Construction of *Streptomyces coelicolor* M1146 mutants containing the GE2270 biosynthetic gene cluster

The original cosmid 2F7 was identified from a SuperCos3 based cosmid library; the vector backbone contains an apramycin and an ampicillin resistance for antibiotic selection [15]. The insert covers the GE2270 biosynthetic gene cluster (*pbt*) from the source strain *Planobispora rosea* ATCC 53733. Additionally it contains the resistance gene EF-Tu and 25 additional ribosomal genes downstream of the cluster, as well as a gene coding for the ß′-subunit of DNA dependent RNA polymerase (*rpoC*) upstream of the cluster (GenBank accession number KF366381.2).

In a first step the apramycin resistance gene (*aac(3)IV*) on the SuperCos3 backbone was exchanged with a chloramphenicol resistance gene (*cat*) from pACYC184 [37]. This was necessary as most planned modifications of cosmid 2F7 were based on cassettes containing an apramycin resistance gene. The *cat* gene was amplified with primer pair targcat_fwd (*TGGGCTACGTCTTGCTGGCGTTCGTTTTCCGGATCGCG* ***A*** **CTAGT**CACTAAATCAGTAAGTTGGC) and targcat_rev (*CGCGACCTTGCCCCTCCAACGTCATCTCGTTCTCCGCTC* **ACTAGT**ATTATCACTTATTCAGGCG), both with *Spe*I restriction sites marked in bold. Italic letters indicate 39 nucleotides homologous to cosmid 2F7 sequences (as for the rest of the described primers) according to the following Red/ET-mediated recombination [38] resulting in cosmid 2F7cat, which was confirmed by restriction analysis. To construct cosmid pbtKA01 the *aac*(3)*IV-tcp830* cassette derived from pMS80 [25] was amplified with primer pair targC/D_fwd (*GAAGAGATCACCCGCGACATCCCCAACGTCTCCGAGGAG* GTGTAGGCTGGAGCTGCTTC) and targC_rev (*TCGGACCACTCGTTCGGCATTACTGCGCGAGCTCGTCAC* TCTAGACCTCCGACGTACGC). The resulting PCR product was utilized in Red/ET mediated recombination to replace the first 5418 bp of the insert of cosmid 2F7cat, consisting mainly of the coding sequence for RNA polymerase subunit β′, and thus placing the *tcp830* promoter in front of *pbtR*. For the construction of pbtKA02 primer targC/D_fwd was reused in combination with primer targD_rev (*CCTCGAAACCGGAGAAAGATATGCCGGCGCCACGGTCAT* TCTAGACCTCCGACGTACGC) to replace the first 6128 bp of the insert of cosmid 2F7cat by Red/ET mediated recombination. Thus, the *tcp830* promoter was placed upstream of the first gene involved in the biosynthesis of GE2270, *pbtG1*, in the same step deleting the transcriptional regulator *pbtR*. Both cosmids were confirmed via restriction analysis and partial sequencing.

For the construction of cosmid pbtCK01 the apramycin resistance cassette [*aac*(3)*IV*] on plasmid pIJ774 was amplified [39] using the primer pair targA-rib_fwd (*ACCGTCGGCGCCGGCCGCGTCACCAAGATCCTCAAGTAG* **GTATAC**ATTCCGGGGATCCGTCGACC) including a restriction site for *Bst*Z171 (bold) and targA-rib_rev (*GACCAGGATCTCCTCGTCCGCGTAGAAGGTGATGAGCGG* **TTATAA**TGTAGGCTGGAGCTGCTTCG) with a restriction site for *Psi*I (bold). The resulting PCR product replaced 12,354 bp in cosmid 2F7cat via Red/ET-mediated recombination [38], comprising 22 ribosomal genes between the *tuf^R^* gene encoding EF-Tu and the SuperCos3 vector. The resulting cosmid pbtCK01 was confirmed by restriction analysis and partial sequencing.

To obtain cosmid pbtCK02, first the apramycin resistance cassette was removed from pbtCK01 *in vitro* by application of Cre recombinase (New England Biolabs, Frankfurt am Main, Germany) according to the manufacturer's manual utilizing the *loxP* recognition sites flanking the apramycin resistance cassette [40]. The correct excision of the cassette was proven by restriction analysis and PCR. In order to insert the constitutive ermE* promoter in front of the *tuf^R^* gene, a 1780-bp fragment was amplified from a previously published pUWL201 derivative [23], [41], using primer pair targB-rib_ermE_f (*GGCAACTCGCTCATCGAGTGCGTGGTCACCGGGGGCTGA* **GTATAC**TCAGGCGCCGGGGGCGGTG) including a restriction site for *Bst*Z171 (bold) and targB-rib_ermE_r (*TGGTCCGCTCGAACTTGGCCTTGGCCACTGTCTGTCTCC* **TTATAA**ATCCTACCAACCGGCACGATTG) including a restriction site for *PsiI* (bold). This PCR- product contained the ermE* promoter and the hygromycin resistance gene (*hyg*) and was inserted into the derivative of cosmid pbtCK01 lacking the [*aac*(3)*IV*] cassette by Red/ET-mediated recombination. This resulted in the loss of 3560 bp, containing the ribosomal genes *rpsL*, *rpsG* and *fusA*, and in placing the *tuf* gene under control of the ermE* promoter. Restriction analysis and partial sequencing verified the accuracy of pbtCK02.

Cosmids pbtCK03, pbtCK04 and pbtCK05 are derivatives of pbtCK02, containing the synthetic tetracycline-inducible promoter *tcp830* from plasmid pMS80 [25]. To construct cosmid pbtCK03(apra) the [*aac*(3)*IV-tcp830*] cassette was amplified and utilized in Red/ET mediated recombination as described for pbtKA01, but targeting the insert of cosmid pbtCK02, thus placing the *tcp830* promoter again in front of *pbtR*. In the same way the construction of pbtCK04(apra) was done as described for pbtKA02, but targeting cosmid pbtCK02 instead of 2F7cat. A third primer pair, targE_fwd (*GTCGACCTGTACGCCGCGACCGAGCAGATCGGCGGATGA* GTGTAGGCTGGAGCTGCTTC) and targE_rev (*CATGGGCAGGTCGTTAAGGTTCAACTCCATCTCGCTCAT* TCTAGACCTCCGACGTACGC), was used to construct pbtCK05(apra) by amplifying the [*aac*(3)*IV*-*tcp830*] cassette for its insertion in front of *pbtA*, coding for the precursor peptide of GE2270, under loss of the 125-bp intergenic region between *pbtM2* and *pbtA*. After successful construction of cosmids pbtCK03(apra), pbtCK04(apra) and pbtCK05(apra) the apramycin resistance cassette was removed from each of them via FLP-mediated excision [42], resulting in cosmids pbtCK03, pbtCK04 and pbtCK05. All three cosmids were confirmed via restriction analysis and partial sequencing.

In addition a negative control cosmid was constructed, which is lacking the entire *pbt* gene cluster but retains the ribosomal genes. For that purpose the apramycin resistance cassette [*aac*(3)*IV*] from plasmid pIJ774 [39] was amplified with primer pair targZ_fwd (*GAAGAGATCACCCGCGACATCCCCAACGTCTCCGAGGAG* ATTCCGGGGATCCGTCGACC) and targZ_rev (*CCCCCGTCGCGAACCCCGGAAGGAGCCGCCGGGCAGGCC* TGTAGGCTGGAGCTGCTTCG). The 1,441-bp PCR fragment was inserted into 2F7cat by Red/ET-mediated recombination replacing the 26,831 bp comprising the entire GE2270 gene cluster.

Cosmids pbtCK01-pbtCK05 and pbtCK08 were transformed into non-methylating *E. coli* ET12567/pUB307 [43] and introduced into *S. coelicolor* M1146 by conjugation [27].

### Agar diffusion test

Approximately 10^5^ spores of *S. coelicolor* M1146, *S. coelicolor* M1146(pbtCK01) and S. *coelicolor* M1146(pbtCK02) were streaked on 20 ml DNA agar plates (0.46 g Difco Nutrient Agar + 20 ml distilled water). GE2270 standard solved in acetonitrile: water (70∶30) was spotted on the plates in final amounts of 0.4 µg, 4 µg, 8 µg and 12 µg. 20 µg Kanamycin were used as positive control, GE2270 solvent was used as negative control, respectively. Inhibition zones were analyzed after 48 hours of cultivation at 30°C.

### Construction of the phylogenetic tree

A neighbor-joining cladogram was constructed with MEGA5 [44]. Alignment of the sequences was done with ClustalW, bootstrap values (in percent) are calculated from 1000 resamplings. The tree was rooted to the 16S rRNA sequence from *Bacillus cereus* ATCC 14579 (NR_074540.1).

16S rRNA sequences of selected actinomycetes were obtained from GenBank, despite *Streptomyces coelicolor* A3(2) (http://strepdb.streptomyces.org.uk) and *Planobispora rosea* ATCC 53733 (Naicons, see Supporting Information S1), *Catenulispora acidiphila* DSM 44928 (accession number (acc. No.): NR_074457.1), *Micromonospora pallida* DSM 43817 (acc. No.: NR_044884.1), *Nocardia aobensis* IFM 0372 (acc. No.: AB126876.1), *Nonomuraea* sp. ATCC 39727 (acc. No.: AJ582011.2), *Propionibacterium acnes* DSM 1897 (acc. No.: X53218.1), *Saccharopolyspora erythraea* NRRL 2338 (acc. No.: NR_074095), *Salinispora tropica* CNB-440 (acc. No.: NR_074502.1), *Streptomonospora salina* YIM90002 (acc. No.: NR_025042.1) and *Streptomyces lividans* NBRC 15678 (acc. No.: AB184694.1).

## Results

### Failure of conjugative transfer of the GE2270 biosynthetic gene cluster with flanking ribosomal genes into *S. coelicolor* M1146

**Figure 2:**
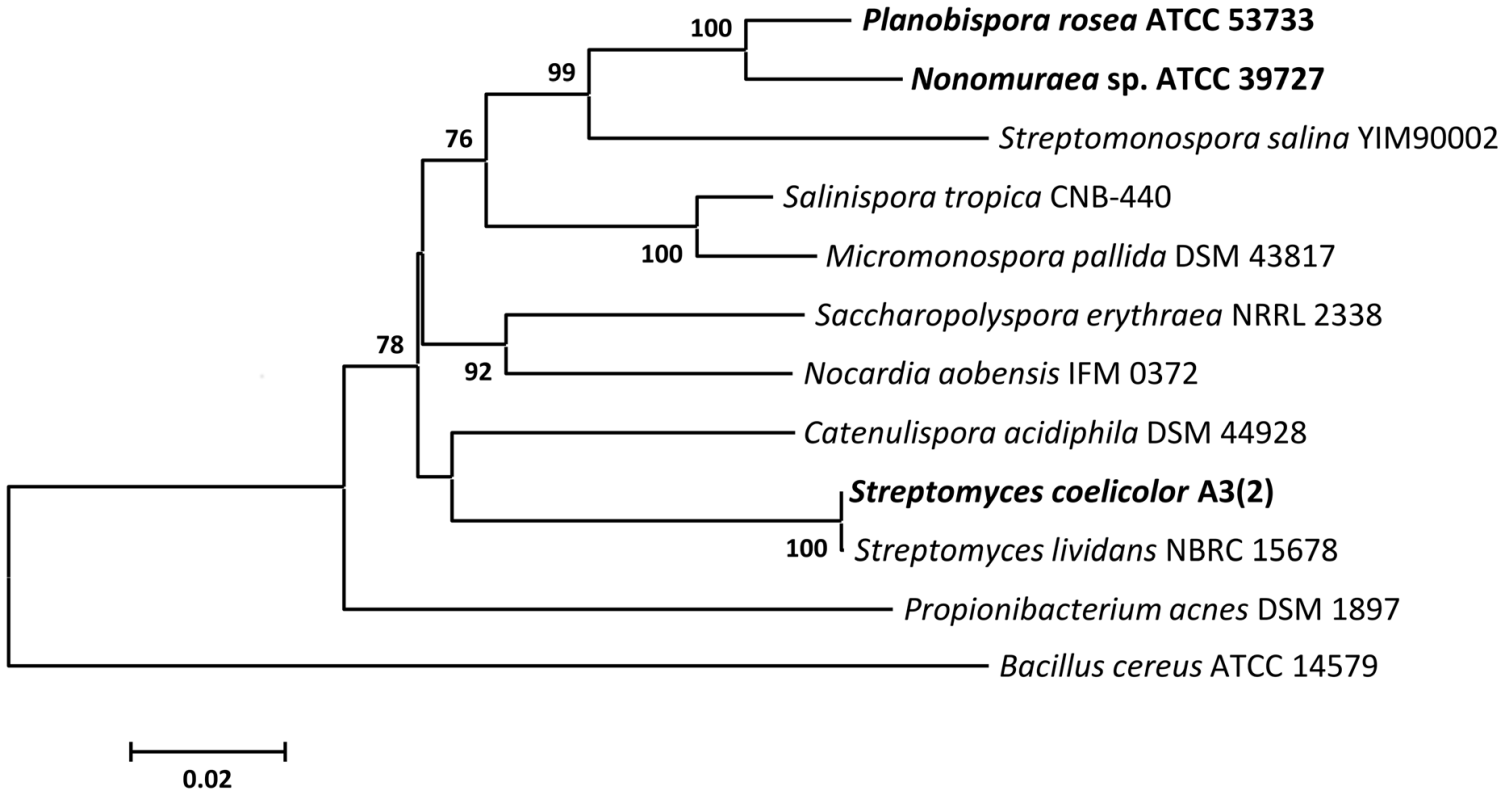
Phylogenetic tree based on 16S rRNA gene sequences of selected actinomycetes. Neighbor-joining cladogram constructed in MEGA5 [44]; alignment of the sequences was done with CLUSTALW, bootstrap values (in percent) are calculated from 1000 resamplings and shown at the respective nodes for values >50%, the scale bar represents 0.02% sequence divergence. The tree is rooted to 16S rRNA from *Bacillus cereus* ATCC 14579.

We focused our interest on the obvious difference between the successful heterologous expression of GE2270 in *Nonomuraea* sp. ATCC 39727 and the unsuccessful attempts in *S. lividans* and *S. albus* observed by Tocchetti *et al.* [15]. As all experiments in this study were based on the same cosmid (2F7), we concluded that the cluster was intact and that the unsuccessful heterologous expression might be due to a lack of transcription of the GE2270 biosynthetic gene cluster (*pbt*) in *Streptomyces*. This lack of transcription could be due to the phylogenetic relationship of the species; *Nonomuraea* sp. ATCC 39727 and *Planobispora rosea* are two closely related species, whereas Streptomycetes are a more distantly related genus of Actinomycetes (Figure 2).

**Figure 3:**
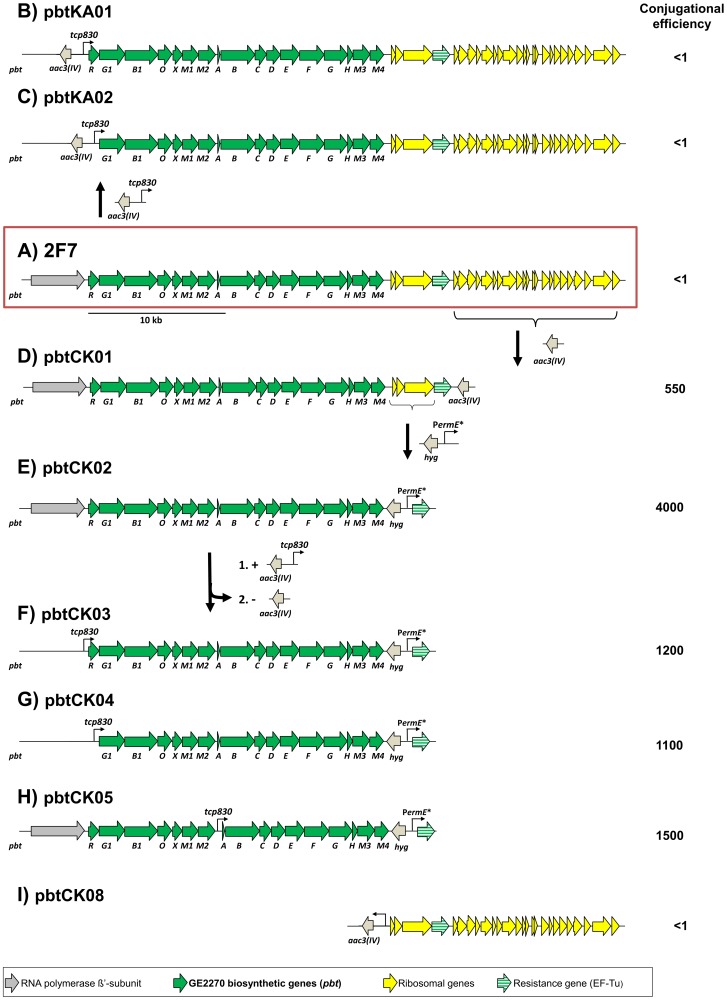
Insert of cosmid 2F7 and construction of cosmids derived from 2F7. A) Insert of 2F7 and 2F7cat comprising the GE2270 biosynthetic gene cluster (*pbt*), the *tuf^R^* gene coding for the GE2270-resistant EF-Tu, 25 adjacent ribosomal genes and *rpoC* coding for RNA polymerase β′-subunit (see Table S2). B) Introduction of the inducible *tcp830* promoter in front of the regulatory gene *pbtR* resulting in cosmid pbtKA01. C) For the construction of pbtKA02 the *tcp830* promoter was introduced in front of *pbtG1* under loss of *pbtR*. D) Replacement of 22 genes encoding ribosomal proteins with an apramycin resistance cassette (*aac(3)IV*) resulted in cosmid pbtCK01. E) pbtCK02 was constructed by introduction of the constitutive promoter ermE* and associated replacement of the ribosomal genes *rpsL*, *rpsG* and *fusA* with a hygromycin resistance cassette (*hyg*). F–H) Introduction of the inducible *tcp830* promoter at three distinct positions in each case followed by subsequent removal of the employed *aac(3)IV* cassette. F) *tcp830* introduced in front of the regulatory gene *pbtR* resulting in cosmids pbtCK03. G) *tcp830* introduced in front of *pbtG1* under loss of *pbtR* resulting in cosmid pbtCK04 and H) *tcp830* introduced in front of the structural gene *pbt*A resulting in cosmid pbtCK05. I) negative control cosmid pbtCK08 was constructed by replacement of the *pbt* biosynthetic genes of cosmid 2F7cat by an apramycin resistance cassette (*aac(3)IV*). For each construct, the efficiency of conjugal transfer into *Streptomyces coelicolor* M1146 is expressed as number of exconjugants per 1 million spores.

All genes of the *pbt* gene cluster are orientated in the same direction (Figure 3), as it is the case for the novobiocin gene cluster, for which the suitability of the inducible *tcp830* promoter for induction of transcription and thus expression of novobiocin was successfully demonstrated [24]. The *tcp830* promoter cassette derived from pMS80 contains an apramycin resistance gene as selection marker. Therefore, we first exchanged the apramycin resistance gene in the SuperCos3 backbone of cosmid 2F7 [15] for a chloramphenicol resistance gene (*cat*), generating cosmid 2F7cat. Then we constructed two derivatives of this cosmid (Figure 3B and C), one placing the *tcp830* promoter in front of the first gene of the *pbt* cluster, encoding the TetR family regulator PbtR (generating pbtKA01), and a second one placing the tcp830 promoter in front of the first biosynthetic gene of the *pbt* cluster, *pbtG1*, while in the same step deleting the regulatory gene *pbtR* (generating pbtKA02). In order to minimize insert size, we deleted the sequence upstream of the *pbt* cluster, containing the *rpoC* gene encoding the β′-subunit of DNA-dependent RNA polymerase, in both constructs in the same step. Both cosmids were then conjugated into *S. coelicolor* M1146 (see introduction). Unexpectedly, the conjugation of both constructs as well as of the original cosmid 2F7 into *S. coelicolor* M1146 failed and no exconjugants were detectable in repeated experiments (Figure 3B and C), suggesting a principal problem during conjugation rather than of transcription.

### Manipulation of cosmid 2F7cat to facilitate its conjugation and heterologous production of GE2270 in *Streptomyces coelicolor* M1146

The insert sequence of cosmid 2F7cat (and 2F7) covers not only the GE2270 biosynthetic gene cluster (*pbt*) from the source strain *Planobispora rosea* ATCC 53733, but also the resistance gene *tuf^R^*, encoding a resistant EF-Tu, 25 additional ribosomal genes downstream of the cluster, as well as the already described gene *rpoC* coding for the ß′-subunit of DNA dependent RNA polymerase upstream of the cluster.

We speculated that either the toxicity of the heterologously formed GE2270 or a detrimental effect of the 25 ribosomal genes from *P. rosea* (Table S2) might prevent the successful conjugative transfer of this cosmid into *S. coelicolor*, and thus we further modified cosmid 2F7cat.

In order to delete most of the ribosomal genes which might interfere with the translational machinery of the expression host, the 22 ribosomal genes downstream of the *tuf^R^* gene were replaced by an apramycin resistance cassette, resulting in cosmid pbtCK01 (Figure 3D). The applied apramycin resistance cassette obtained from pIJ774 [39] is flanked by *loxP* sites enabling subsequent *in vitro* removal of the cassette with the help of Cre recombinase.

To ensure expression of the GE2270 resistance gene *tuf^R^* in the heterologous expression strain *S. coelicolor* M1146, the strong constitutive ermE* promoter [23], [45] was introduced in front of the *tuf^R^* gene (Figure 3E) resulting in cosmid pbtCK02. The last three remaining ribosomal genes *rpsL*, *rpsG* and *fusA* were eliminated at the same time.

To provide unequivocal evidence whether or not the ribosomal genes of *P. rosea* are responsible for the failure of the conjugative transfer of cosmid 2F7 and its derivatives shown in Figure 3B and C into *S. coelicolor*, a further cosmid termed pbtCK08 was constructed, which contained only the 26 ribosomal genes, but not the *pbt* genes encoding GE2270 biosynthesis (Figure 3I).

To ensure transcription of the *pbt* gene cluster in *S. coelicolor* M1146 three additional constructs (pbtCK03, pbtCK04 and pbtCK05) were generated by introducing the tetracycline inducible promoter *tcp830* into distinct positions of the GE2270 gene cluster in cosmid pbtCK02, namely: (i) upstream of the first gene of the cluster, which is the putative regulatory gene *pbtR*, belonging to the *tetR* family of transcriptional regulators, which mostly act as transcriptional repressors [46] (pbtCK03, see Figure 3F); (ii) in front of the second gene of the cluster, *pbtG1*, under simultaneous deletion of the putative transcriptional repressor *pbtR* (pbtCK04, see Figure 3G); (iii) in front of *pbtA*, the structural gene encoding the precursor peptide of GE2270 (see Figure 3H).

### Influence of ribosomal genes from *P. rosea* on conjugation efficiency in *S. coelicolor* M1146

We introduced the seven cosmids depicted in Figure 3A, D-I into *S. coelicolor* M1146 via conjugation as described by Gust *et al*. [38]. For each construct the number of exconjugants was determined from three replicate experiments. Conjugation efficiency was calculated as the number of exconjugants per 10^6^ recipient spores and is given for each cosmid in Figure 3.

As observed previously, the conjugal transfer of cosmid 2F7 was unsuccessful. However, after deletion of the 22 ribosomal genes downstream of the *tuf^R^* gene in cosmid pbtCK01 (Figure 3D) exconjugants were readily obtained (550 per 10^6^ spores). Thus the unsuccessful conjugation of cosmid 2F7 was not caused by a lack of resistance to GE2270 as assumed initially, but indeed seemed to be caused by a conflict of *S. coelicolor* with the ribosomal genes of *P. rosea*. After additional deletion of the remaining three ribosomal genes *rpsL*, *rpsG* and *fusA* and introduction of the ermE* promoter (cosmid pbtCK02, Figure 3E), conjugation efficiency increased again 7 to 8-fold; reaching 4000 exconjugants per 10^6^ spores. As expected, similar results were obtained with cosmids pbtCK03-05, depicted in Figure 3F, G and H.

Cosmid pbtCK08, containing all ribosomal genes, but lacking the GE2270 biosynthetic gene cluster proved to be as unsuitable for conjugal transfer as the original unmodified cosmid 2F7. This confirms that the ribosomal genes flanking the *pbt* gene cluster are indeed the reason for the unsuccessful attempts at conjugation of cosmid 2F7 into *S. coelicolor*.

### Media screen and heterologous production of GE2270A in *S. coelicolor* M1146(pbtCK01)

For each of the five cosmids transferred efficiently into *S. coelicolor* M1146, three exconjugants were tested for production of GE2270 using published production media for thiazolylpeptides, namely the “main production medium” described by Selva *et al*. [29], RARE3 [30], Fermentation medium C [31], M8 [32], GE37468 production medium [13] and AF medium [33]. Anhydrotetracycline (aTc) was added to induce the *tcp830* promoter, where required. However, no production of GE2270 congeners could be detected via HPLC and LC-MS in any of these exconjugants, whereas GE2270 production was detected readily in each of these media in the native producer *P. rosea*. To proof sample treatment and analytical methods, the HPLC detection limit was ascertained to be at 0.02 µg corresponding to a production rate of 0.08 mg l^−1^. Extraction of prior added GE2270A standard to the culture resulted in a recovery rate of 73%.

To investigate whether the *pbt* gene cluster on cosmid pbtCK01 was still intact, we conjugated this cosmid into *Nonomuraea* sp. ATCC 39727 and compared its production to *Nonomuraea* sp. ATCC 39727 containing cosmid 2F7. Both strains produced comparable amounts of GE2270A over time, demonstrating the GE2270 cluster on cosmid pbtCK01 to be fully intact (Figure S1).

**Figure 4:**
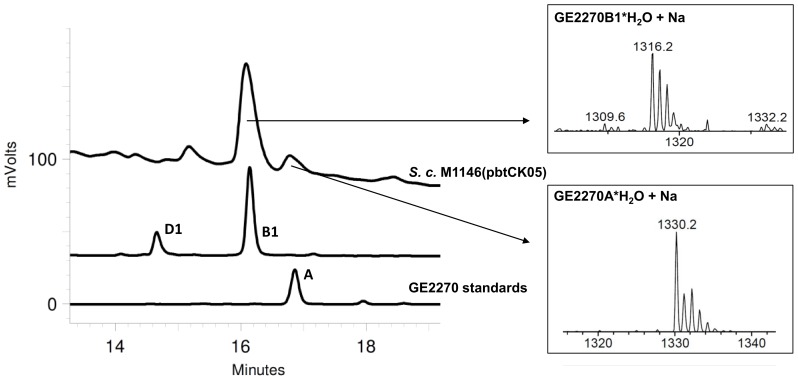
HPLC-MS analysis of a methanolic extract of *S. coelicolor* M1146(pbtCK05). Data confirms the presence of GE2270A; GE2270B1 is identified as the main product.

Since no GE2270 congeners were produced by the heterologous expression strains in previously published thiopeptide production media, we decided to investigate GE2270 production in 35 additional production media, 31 derived from a screen media list kindly provided by EntreChem, S. L. (Oviedo, Spain), as well as four media previously employed in our laboratory for antibiotic production in *S. coelicolor* M1146, i.e. chemically defined medium (CDM) described by Kominek [34], MS medium, [27] GYM medium [35] and phenazine production medium [36]. *S. coelicolor* M1146(pbtCK01) was cultivated in each of these media. Three replicate cultures were prepared for each medium using 24-square deepwell plates. After 3 and 6 days of cultivation culture extracts were analyzed via HPLC. GE2270 production was detected only in a single medium out of the investigated 35 media, namely in screening medium 13 (SM13), originally described by Imada *et al.* [47] and named “main fermentation medium”. The presence and identity of at least two GE2270 congeners, GE2270A and GE2270B1, was confirmed by HPLC-MS (see Figure 4) with a mean production of GE2270A of about 1 mg l^−1^. The ingredients of this medium are glucose, glycerol, Pharmamedia®, soluble starch, corn steep liquor, CaCO_3_, peptone and NaCl. All of these ingredients are also contained in some of the other screened media, thus it was not possible to conclude why only this medium and none of the others gave rise to GE2270 production in *S. coelicolor* M1146.

Addition of 0.6% of the water-soluble siloxylated ethylene oxide/propylene oxide copolymer Q2-5247 (Dow Corning, USA), suggested to act as oxygen carrier, has been shown to increase secondary metabolite production in some heterologous expression strains [22], [48]. However, it had no influence on GE2270 production (Data not shown).

**Figure 5:**
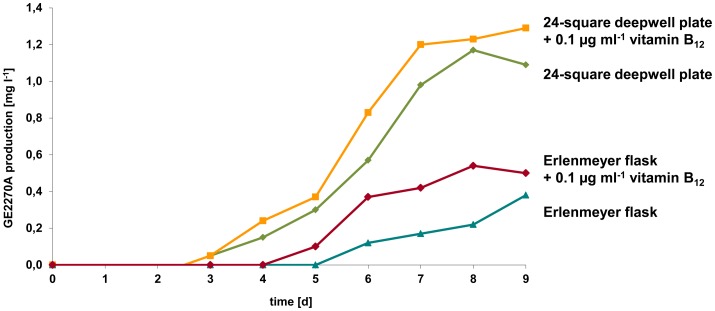
GE2270A production over time in *S. coelicolor* M1146(pbtCK01). Cultivation was performed in SM13 medium in 300-square deepwell plates [28].

Addition of vitamin B_12_ (cobalamin) during cultivation of the natural producer strain *P. rosea* [31] has been reported to lead to a doubling of total GE2270 complex production and to shift the relative composition towards the most methylated compound, GE2270A. Therefore we added vitamin B_12_ (0.1 µg ml^−1^) to SM13 medium and analyzed GE2270 production in 24-square deepwell plates and Erlenmeyer flasks (Figure 5). GE2270A production is nearly 3-fold higher in 24-square deepwell plates than in Erlenmeyer flasks. However, supplementation of vitamin B_12_ resulted only in a slightly higher formation of the completely methylated congener, GE2270A.

### Influence of constitutive expression of the resistance gene and of introduction of the *tcp*830 promoter at different positions of the cluster on heterologous production of GE2270

**Figure 6:**
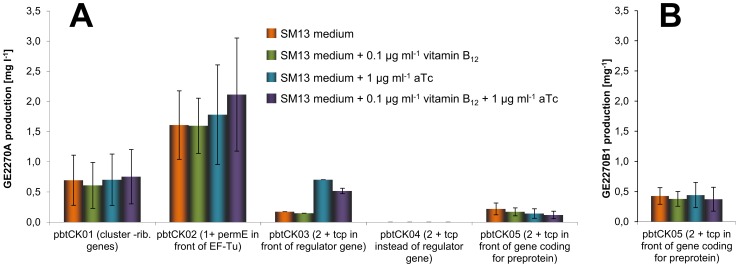
GE2270 production of S. coelicolor M1146 strains containing different constructs. A) GE2270A production of S. *coelicolor* M1146 strains containing different constructs of the GE2270 biosynthetic gene cluster (see Figure 3). The amount of GE2270A was determined after 8 days of cultivation in 24-square deepwell plates [28]. B) GE2270B1 production in S. *coelicolor* M1146(pbtCK05). Values are mean ± SEM from triplicated cultivation of three individual exconjugants each.

After establishing heterologous expression of GE2270 in *S. coelicolor* M1146(pbtCK01), we examined GE2270 production resulting from expression of the modified *pbt* cosmids shown in Figure 3E, F, G and H. All strains were grown in SM13 medium using 24-square deepwell plates and cultivating three independent exconjugants for each construct. Cultivation was carried out with and without addition of anhydrotetracycline (aTc), which induces transcription from the *tcp830* promoter [24], [25], and with and without addition of vitamin B_12_. The results are depicted in Figure 6.

Cultivating *S. coelicolor* M1146(pbtCK01), GE2270A production was approximately 0.7 mg l^−1^ under all tested conditions (Figure 6A). As expected, addition of aTc did not have any effect on GE2270 production, as there is no inducible promoter present in this construct.

In *S. coelicolor* M1146(pbtCK02), in which the constitutive ermE* promoter has been inserted in front of the resistance gene *tuf^R^*, coding for the GE2270 insensitive EF-Tu, production was 2.5-fold higher compared to *S. coelicolor*M1146(pbtCK01) under all measured conditions (Figure 6A).

Insertion of the inducible *tcp830* promoter upstream of the gene cluster (pbtCK03, Figure 3F) resulted in very low GE2270A production (0.17 mg l^−1^) when the heterologous expression strain was cultivated without aTc, which was to be expected, as the tcp830 promoter is not fully repressed in *S. coelicolor* [25]. However, when the inducer aTc was added, production increased about 4-fold to 0.7 mg l^−1^, but remained lower than in *S. coelicolor* M1146(pbtCK02) (Figure 6A).

Deletion of *pbtR*, assumed to encode a transcriptional repressor belonging to the TetR-family, together with a placement of the first biosynthetic gene *pbtG1* under control of the *tcp830* promoter (*S. coelicolor* M1146(pbtCK04)), unexpectedly resulted in a complete abolishment of GE2270 biosynthesis (Figure 6A). This suggests that PbtR, though belonging to a family known as transcriptional repressors, is strictly needed for GE2270 production.

In the last construct the *tcp830* promoter was introduced in front of the structural gene *pbtA*, encoding the precursor peptide of GE2270 (*S. coelicolor* M1146(pbtCK05)). Surprisingly, production of GE2270A was not inducible with aTc and, with an average of 0.14 mg l^−1^, corresponding to the amount detected in *S. coelicolor* M1146(pbtCK03) without induction. In contrast to all other strains, GE2270A was not the main congener produced by this strain. Another GE2270 derivative was detected to accumulate in the extracts in amounts of 0.5 mg l^−1^ (Figure 6B). HPLC-MS confirmed this main product to be GE2270B1, a congener lacking a N-methyl group (Figures 1 and 4). Gene *pbtM1* encodes the N-methyltransferase likely responsible for the conversion of GE2270B1 into GE2270A and is situated upstream of the *tcp830* promoter in this construct. Production of GE2270B1 was not induced by addition of aTc.

### GE2270A resistance of *S. coelicolor* M1146 and derivatives

**Figure 7:**
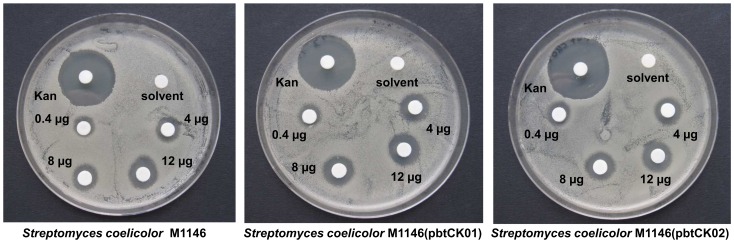
Agar diffusion test to determine the resistance of *Streptomyces coelicolor* strains against GE2270A. GE2270A was applied in amounts of 0.4 µg to 12 µg; 20 µg kanamycin (Kan) were applied as positive control.

To clarify whether the resistant EF-Tu from *P. rosea* also conveys resistance against GE2270A to the heterologous producer strains, an agar diffusion test was carried out. Resistance of *S. coelicolor* M1146, *S. coelicolor* M1146(pbtCK01) and *S. coelicolor* M1146(pbtCK02) against GE2270A was investigated. After two days of cultivation, growth inhibition was clearly visible for all three strains, even at the lowest amount of 0.4 µg (Figure 7), proving that they are equally sensitive against GE2270A. No relevant difference was observable between *S. coelicolor* M1146, not carrying the resistance gene *tuf^R^* of *P. rosea*, *S. coelicolor* M1146(pbtCK01) carrying the *tuf^R^* gene coding for the resistant EF-Tu under control of its own promoter and *S. coelicolor* M1146(pbtCK02) which carries the *tuf^R^* gene under control of the constitutive ermE* promoter. Therefore, *tuf^R^* which has been proven to convey GE2270 resistance to *P. rosea* [49], [50] does not convey significant resistance to *S. coelicolor*, and obviously this may limit GE2270 production rates in the heterologous expression strain.

## Discussion

In this study we successfully solved the problem of conjugative transfer of the GE2270 biosynthetic gene cluster into Streptomycetes, allowing for the first time heterologous production of GE2270 in a *Streptomyces* strain. We thereby demonstrated that genes belonging to primary metabolism of phylogenetic distant donor species may prevent conjugal transfer of the desired biosynthetic gene cluster.

Our first attempt to achieve heterologous production of GE2270 in *S. coelicolor* M1146 targeted the transcription of the *pbt* cluster by introduction of the inducible *tcp830* promoter, which has been used very successfully to induce heterologous production of novobiocin in *S. coelicolor* [24]. Two new cosmids were constructed, placing either the regulatory gene *pbtR* (pbtKA01, Figure 3B) or the first of the biosynthetic genes (pbtKA02, Figure 3C) under control of the *tcp830* promoter. Neither the two constructs nor the original cosmid 2F7 could be conjugated into *S. coelicolor* M1146. These results indicated a possible toxicity issue caused either by a toxic effect of GE2270 on *S. coelicolor* M1146, or by a detrimental effect of the ribosomal genes of *P. rosea*, which are contained in these cosmids.

To solve this problem, two new cosmids were constructed, one by deleting most of the ribosomal genes (pbtCK01, Figure 3D) and the other by placing the resistance gene *tuf^R^* under control of the constitutive ermE* promoter, at the same time deleting the remaining three ribosomal genes (pbtCK02, Figure 3E). Interestingly, we achieved normal amounts of exconjugants in *S. coelicolor* M1146 already with cosmid pbtCK01, after deletion of most of the ribosomal genes. The number of exconjugants was further increased with cosmid pbtCK02, probably due to the deletion of the three remaining ribosomal genes rather than the constitutive expression of *tuf^R^*, as production of GE2270 was only detected in screening medium 13. As expected, similar conjugation efficiencies could be obtained for the cosmids pbtCK03, pbtCK04 and pbtCK05 (Figure 3F-H), but not with cosmid pbtCK08 (Figure 3I), which lacks the entire biosynthetic gene cluster, but still contains all ribosomal genes. In contrast, conjugation of 2F7 and pbtCK01 into *Nonomuraea* sp. ATCC 39727, a strain closely related to *Planobispora rosea* (Figure 2), was successful. This demonstrates that indeed the ribosomal genes on cosmid 2F7 are responsible for its detrimental effect and prevent conjugation into *Streptomyces*, but not into *Nonomuraea* sp. ATCC 39727. In this heterologous producer no differences in GE2270A production were visible, whether the original cosmid 2F7 or pbtCK01 was integrated into its genome (Figure S1). The greater tolerance towards the foreign ribosomal genes observed by *Nonomuraea* sp. ATCC 39727 seems to be based on the closer phylogenetic relationship of the two strains. Possibly, these foreign ribosomal genes impair formation of functional ribosomes in exconjugants of *S. coelicolor* and render them non-viable.

In contrast, exconjugants obtained from *S. coelicolor* M1146 with the modified cosmids pbtCK01 and pbtCK02 were clearly viable, but nonetheless GE2270 production could not be detected in previously described thiopeptide production media. Eventually, we detected GE2270 production in only a single medium out of 41. This emphasizes the importance of testing a variety of media in heterologous expression experiments. Production in the *Streptomyces* host was lower (max. 3 mg l^−1^ GE2270A under optimal conditions using pbtCK02) than in the native producer *P. rosea* or in the heterologous producer *Nonomuraea* sp. ATCC 39727. A similar observation has been reported for the heterologous expression of the related thiopeptide GE37468 from *Streptomyces* ATCC 55365 in *Streptomyces lividans*, where production only reached 2–3 mg l^−1^ [13].

Notably, a 2.5-fold higher GE2270 production was observed using pbtCK02 as compared to pbtCK01. This increase might be due to the deletion of the remaining three ribosomal genes in pbtCK02 rather than to the constitutive expression of the resistance gene *tuf^R^*.

In agar diffusion tests *S. coelicolor* M1146, as well as the strains *S. coelicolor* M1146(pbtCK01) and *S. coelicolor* M1146(pbtCK02) were sensitive to GE2270A; despite of the fact that the latter two strains contain the resistance gene *tuf^R^* [49] under control of either its native promoter or the constitutive ermE* promoter. Thus, the resistance gene *tuf^R^* is apparently not able to confer full resistance against GE2270 to the heterologous host strain *S. coelicolor* M1146. Elongation factor Tu is a very abundant protein, playing a central role in ribosomal protein synthesis by delivering aminoacyl-tRNA to the growing protein chain. Possibly, the efficiency of the heterologous, GE2270-resistant EF-Tu for protein biosynthesis in *S. coelicolor* is quite low, or its level of expression is too low to confer full resistance.

In this study, we also tried to achieve inducible GE2270 production by introducing the tetracycline-inducible *tcp830* promoter [25]. Application of this promoter/inducer system has been shown to be free of unwanted pleiotropic effects [25]. The insertion of *tcp830* upstream of the entire gene cluster indeed results in an aTc inducible 4-fold increase in GE2270 production. Placement of the *tcp830* promoter in front of the structural gene *pbtA* in construct pbtCK05 leads to the formation of GE2270B1 as main product but also to smaller amounts of GE2270A. This can be explained by the organization of the *pbt* gene cluster, where some of the responsible genes for the biosynthesis of the final product, GE2270A, are located upstream of the introduced promoter and thus GE2270B1 is accumulated as an intermediate in GE2270A biosynthesis. Surprisingly, GE2270 production was not inducible by aTc in this construct. In *S. coelicolor* M1146(pbtCK04) in addition to the insertion of the *tcp830* promoter, *pbtR*, the regulator gene of the cluster, was deleted. This resulted in a total abolishment of GE2270 production. PbtR is a TetR-like protein with 64% sequence identity to TpdR, the transcriptional regulator of the *tpd* cluster from the thiomuracin- and GE2270-producer strain *Nonomuraea sp.* WU8817 [18]. In that study quantitative RT-PCR showed a strong induction of expression of *tpdR* and *tpdA* (the homolog of *pbtA*) in correlation with the onset of antibiotic production. This observation, in combination with the abolishment of GE2270 production in construct pbtCK04 shown in the present study, suggests a role as positive regulator for PbtR. While most TetR-family regulators act as transcriptional repressors, there are some which activate transcription [46].

Neither of our three constructs improved GE2270 production to levels similar or even higher to those observed for *S. coelicolor* M1146(pbtCK02). In conjunction with the missing inducibility of pbtCK05, this suggests a more complex regulation of the GE2270 biosynthetic gene cluster by more than one promoter region.

In conclusion, in this study we identified and solved a principal problem in the heterologous expression of the GE2270 biosynthetic gene cluster in *Streptomyces.* It was shown that genes belonging to the primary metabolism, in our case the ribosomal genes flanking the *pbt* gene cluster, can completely prevent the success of heterologous expression experiments. This may be of considerable importance for future studies, especially studies using recently developed methods for heterologous expression of large secondary metabolite gene clusters, e.g. by use of P1-derived artificial chromosome (PAC) vectors, which can tolerate inserts up to a size of approximately 200 kb [51].

## Supporting Information

Figure S1 **Comparison of the original cosmid 2F7 and pbtCK01 lacking 22 ribosomal genes concerning GE2270A production over time in** ***Nonomuraea*** **sp. ATCC 39727.**
(PDF)

Supporting Information S1 **Sequence of 16S rRNA of** ***Planobispora rosea*** **ATCC 53733.**
(DOCX)

Table S1 **List of strains, plasmids and cosmids employed in this study.**
(PDF)

Table S2 **List of ribosomal proteins contained in cosmid 2F7 (GenBank accession number KF366381.2) compared to their orthologous in** ***S. coelicolor*** **A3(2).**
(DOCX)
