# Supplementary material for: Heterologous Expression of the Thiopeptide Antibiotic GE2270 from Planobispora rosea ATCC 53733 in Streptomyces coelicolor Requires Deletion of Ribosomal Genes from the Expression Construct
Source: PLoS One. 2014 Mar 5;9(3):e90499. doi: 10.1371/journal.pone.0090499 (PMC3943966; doi:10.1371/journal.pone.0090499)
Supplement: Figure S1 — Comparison of the original cosmid 2F7 and pbtCK01 lacking 22 ribosomal genes concerning GE2270A production over time in Nonomuraea sp. ATCC 39727. (PDF) [file pone.0090499.s001.pdf]

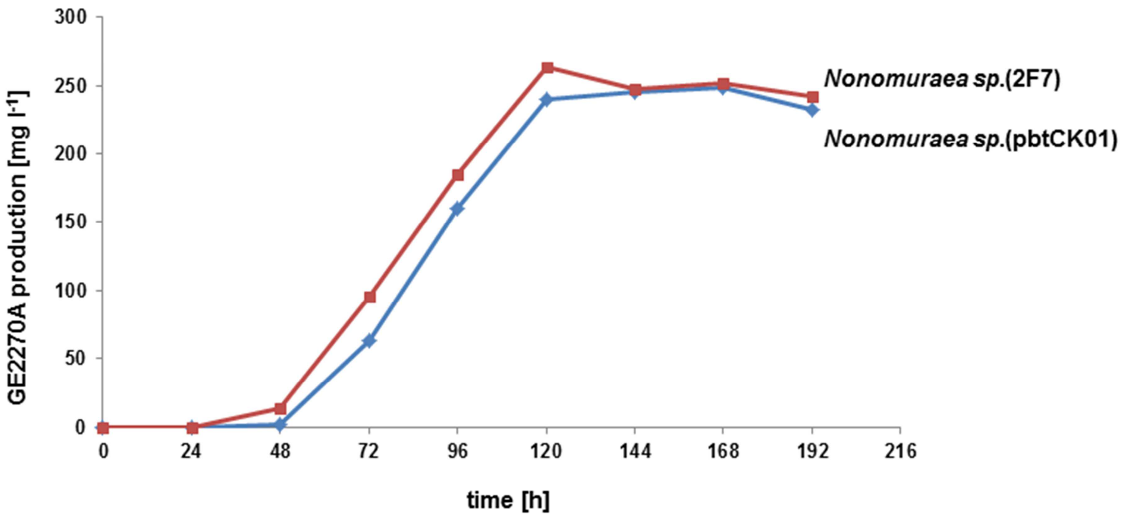

Figure S1. Comparison of the original cosmid 2F7 and pbtCK01 lacking 22 ribosomal genes concerning GE2270A production over time in *Nonomuraea* sp. ATCC39727.
