# Supplementary material for: Heterologous Expression of the Thiopeptide Antibiotic GE2270 from Planobispora rosea ATCC 53733 in Streptomyces coelicolor Requires Deletion of Ribosomal Genes from the Expression Construct
Source: PLoS One. 2014 Mar 5;9(3):e90499. doi: 10.1371/journal.pone.0090499 (PMC3943966; doi:10.1371/journal.pone.0090499)
Supplement: Supporting Information S1 — Sequence of 16S rRNA of Planobispora rosea ATCC 53733. (DOCX) [file pone.0090499.s002.docx]

>*Planobispora rosea* ATCC 53733 16S rRNA

AGAGTTTGATCCTGGCTCAGGACGAACGCTGGCGGCGTGCTTAACACATGCAAGTCGAGCGGAAAGGCCCTTCGGGGTACTCGAGCGGCGAACGGGTGAGTAACACGTGAGCAACCTGCCCCTGACTCTGGGATAAGCCTGGGAAACCGGGTCTAATACCGGATACGACCCCTCACCGCATGGTGCGGGGGTGGAAAGTTTTTCGGTTGGGGATGGGCTCGCGGCCTATCAGCTTGTTGGTGAGGTAGTGGCTCACCAAGGCGACGACGGGTAGCCGGCCTGAGAGGGCGACCGGCCACACTGGGACTGAGACACGGCCCAGACTCCTACGGGAGGCAGCAGTGGGGAATATTGCGCAATGGGCGAAAGCCTGACGCAGCGACGCCGCGTGGGGGATGACGGCCTTCGGGTTGTAAACCTCTTTCAGCAGGGACGAAAGTGACGTGTACCTGCAGAAGAAGCGCCGGCTAACTACGTGCCAGCAGCCGCGGTAATACGTAGGGCGCAAGCGTTGTCCGGAATTATTGGGCGTAAAGAGCTCGTAGGTGGCTGGTCGCGTCGGATGTGAAAGCCCGCAGCTTAACTGCGGGTCTGCATTCGATACGGGCCGGCTAGAGGTAGGTAGGGGCAAGCGGAATTCCTGGTGTAGCGGTGAAATGCGCAGATATCAGGAGGAACACCGGTGGCGAAGGCGGCTTGCTGGGCCTTACCTGACGCTGAGGAGCGAAAGCGTGGGGAGCGAACAGGATTAGATACCCTGGTAGTCCACGCTGTAAACGTTGGGCGCTAGGTGTGGGGGCCTTCCACGGTTCCCGTGCCGTAGCTAACGCATTAAGCGCCCCGCCTGGGGAGTACGGCCGCAAGGCTAAAACTCAAAGGAATTGACGGGGGCCCGCACAAGCGGCGGAGCATGTTGCTTAATTCGACGCAACGCGAAGAACCTTACCAAGGCTTGACATCGCCCGGAAAGCTCTAGAGATAGAGCCCTCTTCGGACTGGGTGACAGGTGGTGCATGGCTGTCGTCAGCTCGTGTCGTGAGATGTTGGGTTAAGTCCCGCAACGAGCGCAACCCTTGTTCCATGTTGCCAGCAACATCCTTCGGGGTGGTTGGGGACTCATGGGAGACTGCCGGGGTCAACTCGGAGGAAGGTGGGGATGACGTCAAGTCATCATGCCCCTTATGTCTTGGGCTGCAAACATGCTACAATGGCCGGTACAGAGGGCTGCCATACCGTAAGGTGGAGCGAATCCCTAAAAGCCGGTCTCAGTTCGGATTGGGGTCTGCAACTCGACCCCATGAAGTCGGAGTCGCTAGTAATCGCAGATCAGCAACGCTGCGGTGAATACGTTCCCGGGCCTTGTACACACCGCCCGTCACGTCACGAAAGTCGGCAACACCCGAAGCCCGTGGCCCAACCCGTAAGGGGGGGAGCGGTCGAAGGTGGGGCTGGCGATTGGGACGAAGTCGTAACAAGGTAGCCGTACCGGAAGGTGCGGCTGGATCACCTCCTTTCTAAGGAGCACC

**Supporting Information S1. Sequence of 16S rRNA of *Planobispora rosea* ATCC 53733.**
