# Supplementary material for: Heterologous Expression of the Thiopeptide Antibiotic GE2270 from Planobispora rosea ATCC 53733 in Streptomyces coelicolor Requires Deletion of Ribosomal Genes from the Expression Construct
Source: PLoS One. 2014 Mar 5;9(3):e90499. doi: 10.1371/journal.pone.0090499 (PMC3943966; doi:10.1371/journal.pone.0090499)
Supplement: Table S1 — List of strains, plasmids and cosmids employed in this study. (PDF) [file pone.0090499.s003.pdf]

Table S1. List of strains, plasmids and cosmids employed in this study

Strain, plasmid or cosmid	Description*	Reference and source
Planobispora rosea ATCC 53733	Natural producer strain of GE2270, contains the *pbt* biosynthetic gene cluster	Naicons strain collection [1]
Nonomuraea sp. ATCC 39727	Producer strain of glycopeptide A40926; suitable for heterologous expression of GE2270	Naicons strain collection [2]
S. coelicolor M1146	SCP1$^-$, SCP2$^-$, *Δact, Δred, Δcpk, Δcda*	Gomez-Escribano and Bibb, 2011 [3]
S. coelicolor M1146(pbtKA01)	*S. coelicolor* M1146 containing 2F7 with *tcp830* in front of *pbtR*; Cm^r, Bla^r, Apra^r	This work
S. coelicolor M1146(pbtKA02)	*S. coelicolor* M1146 containing 2F7 with *tcp830* in front of *pbtG1; lacks pbtR*, Cm^r, Bla^r, Apra^r	This work
S. coelicolor M1146(pbtCK01)	*S. coelicolor* M1146 containing the *pbt* gene cluster, lacks 22 ribosomal genes of 2F7; Cm^r, Bla^r, Apra^r	This work
S. coelicolor M1146(pbtCK02)	*S. coelicolor* M1146 containing the *pbt* gene cluster, lacks 25 ribosomal genes of 2F7; *PermE** in front of *tuf*; Cm^r, Bla^r, Hyg^r	This work
S. coelicolor M1146(pbtCK03)	*S. coelicolor* M1146 containing the *pbt* gene cluster, lacks 25 ribosomal genes of 2F7; *PermE** in front of *tuf*; *tcp830* in front of *pbtR*; Cm^r, Bla^r, Hyg^r	This work
S. coelicolor M1146(pbtCK04)	*S. coelicolor* M1146 containing the *pbt* gene cluster, lacks 25 ribosomal genes of 2F7; *PermE** in front of *tuf*; *tcp830* in front of *pbtG1; lacks pbtR*; Cm^r, Bla^r, Hyg^r	This work
S. coelicolor M1146(pbtCK05)	*S. coelicolor* M1146 containing the *pbt* gene cluster, lacks 25 ribosomal genes of 2F7; *PermE** in front of *tuf*; *tcp830* in front of *pbtA*; Cm^r, Bla^r, Hyg^r	This work
S. coelicolor M1146(pbtCK08)	*S. coelicolor* M1146 containing 25 ribosomal genes and *tuf*; lacks *pbt* gene cluster; Cm^r, Bla^r, Apra^r	This work
E. coli BW25113	K-12 BD792 derivative: *lacI*^{q}, *rrnB*$_{T14}$, Δ*lacZ*$_{WJ16}$, *hsdR514*, Δ*araBAD*$_{AH33}$ *ΔrhaBAD*$_{LD78}$	Datsenko and Wanner, 2000 [4]
E. coli BT340	DH5α/pCP20	Cherepanov and Wackernagel, 1995 [5]
E. coli DH5α	*rec*A1, *end*A1, *hsd*R17, g*yr*A96, *rel*A1,	Stratagene
E. coli ET12567	Nonmethylation strain (*dam*$^-$ *dcm*$^-$ *hsdM*$^-$), Tet^R, Cm^R	MacNeil *et al.*, 1992 [6]
**plasmid or cosmid**		
pIJ790	λ-RED *(gam, bet, exo*), *cat, araC, rep101*^{ts}	Gust *et al.*, 2003 [7]
pUB307	RP4 derivative *oriT*$^+$*,* Kan^R	Bennett *et al.*, 1977 [8]<br>Flett *et al.*, 1997 [9]
pIJ774	*aac(3)IV* (Apra^r), *oriT*$^+$*,* loxP	Khodakaramian *et al.*, 2006 [10]
pUWL201*hyg*	*PermE**, *hyg* (Hyg^r), Bla^r, *oriT*$^+$	Saleh *et al.* 2012 [11]
pMS80	Plasmid containing the *aac(3)IV-tcp830* cassette; Apra^R	Rodriguez-Garcia *et al.*, 2005 [12]

2F7	SuperCos3 based, containing GE2270 biosynthetic gene cluster (*pbt*), $Apra^R$, Bla^R	Tocchetti *et al.*, 2013 [13]
2F7cat	2F7, *cat* (Cm^R) instead of $Apra^R$	This work
pbtCK01	2F7cat ribosomal genes replaced by 774	This work
pbtCK02	2F7cat lacking 25 ribosomal genes; *PermE** in front of *tuf*, Cm^R, Hyg^R	This work
pbtCK03	2F7cat lacking 25 ribosomal genes; *PermE** in front of *tuf, tcp830* promoter upstream of *pbtR;* Cm^R, Hyg^R	This work
pbtCK04	2F7cat lacking 25 ribosomal genes; *PermE** in front of *tuf, tcp830* promoter upstream of *pbtG1*, lacking *pbtR;* Cm^R, Hyg^R	This work
pbtCK05	2F7cat lacking 25 ribosomal genes; *PermE** in front of *tuf, tcp830* promoter upstream of *pbtA;* Cm^R, Hyg^R	This work
pbtCK08	2F7cat containing 25 ribosomal genes and *tuf*; lacks *pbt* gene cluster; $Apra^r$	This work

* Cm^R chloramphenicol-, Bla^R ampicillin-, $Apra^r$ apramycin-, Hyg^R hygromycin-, Tet^R tetracyclin-, kan^R kanamycin-resistant

References

1. Selva E, Beretta G, Montanini N, Saddler GS, Gastaldo L, et al. (1991) Antibiotic GE2270 a: a novel inhibitor of bacterial protein synthesis. I. Isolation and characterization. J Antibiot (Tokyo) 44: 693-701.
2. Goldstein BP (1988) Antibiotic A40926 mannosyl aglycon. US Patent 4,782,042.
3. Gomez-Escribano JP, Bibb MJ (2011) Engineering *Streptomyces coelicolor* for heterologous expression of secondary metabolite gene clusters. Microb Biotechnol 4: 207-215.
4. Datsenko KA, Wanner BL (2000) One-step inactivation of chromosomal genes in *Escherichia coli* K-12 using PCR products. Proc Natl Acad Sci USA 97: 6640-6645.
5. Cherepanov PP, Wackernagel W (1995) Gene disruption in *Escherichia coli*: Tc^R and Km^R cassettes with the option of Flp-catalyzed excision of the antibiotic-resistance determinant. Gene 158: 9-14.
6. MacNeil DJ, Gewain KM, Ruby CL, Dezeny G, Gibbons PH, et al. (1992) Analysis of *Streptomyces avermitilis* genes required for avermectin biosynthesis utilizing a novel integration vector. Gene 111: 61-68.
7. Gust B, Challis GL, Fowler K, Kieser T, Chater KF (2003) PCR-targeted *Streptomyces* gene replacement identifies a protein domain needed for biosynthesis of the sesquiterpene soil odor geosmin. Proc Natl Acad Sci U S A 100: 1541-1546.
8. Bennett PM, Grinsted J, Richmond MH (1977) Transposition of *TnA* does not generate deletions. Mol Gen Genet 154: 205-211.
9. Flett F, Mersinias V, Smith CP (1997) High efficiency intergeneric conjugal transfer of plasmid DNA from *Escherichia coli* to methyl DNA-restricting streptomycetes. FEMS Microbiol Lett 155: 223-229.
10. Khodakaramian G, Lissenden S, Gust B, Moir L, Hoskisson PA, et al. (2006) Expression of Cre recombinase during transient phage infection permits efficient marker removal in *Streptomyces*. Nucleic Acids Res 34: e20.
11. Saleh O, Bonitz T, Flinspach K, Kulik A, Burkard N, et al. (2012) Activation of a silent phenazine biosynthetic gene cluster reveals a novel natural product and a new resistance mechanism against phenazines. Med Chem Commun 3: 1009-1019.
12. Rodriguez-Garcia A, Combes P, Perez-Redondo R, Smith MCA, Smith MCM (2005) Natural and synthetic tetracycline-inducible promoters for use in the antibiotic-producing bacteria *Streptomyces*. Nucleic Acids Res 33: e87.
13. Tocchetti A, Maffioli S, Iorio M, Alt S, Mazzei E, et al. (2013) Capturing linear intermediates and C-terminal variants during maturation of the thiopeptide GE2270. Chem Biol 20: 1067-1077.
