# Supplementary material for: Heterologous Expression of the Thiopeptide Antibiotic GE2270 from Planobispora rosea ATCC 53733 in Streptomyces coelicolor Requires Deletion of Ribosomal Genes from the Expression Construct
Source: PLoS One. 2014 Mar 5;9(3):e90499. doi: 10.1371/journal.pone.0090499 (PMC3943966; doi:10.1371/journal.pone.0090499)
Supplement: Table S2 — List of ribosomal proteins contained in cosmid 2F7 (GenBank accession number KF366381.2) compared to their orthologous in S. coelicolor A3(2). (DOCX) [file pone.0090499.s004.docx]

**Table S2. List of ribosomal proteins contained in cosmid 2F7 (GenBank accession number KF366381.2) compared to their orthologous in *S. coelicolor* A3(2)**

| *Planobispora rosea*,  ribosomal proteins  contained in cosmid 2F7 | Size (aa) | Identity (aa, %) compared to *Streptomyces coelicolor* A3(2) |
| --- | --- | --- |
| ribosomal protein S12 (*rpsL*) | 123 | 91 |
| ribosomal protein S7 (*rpsG*) | 156 | 80 |
| translocation factor G (*fusA*) | 697 | 77 |
| EF-Tu (*tuf^R^*) | 397 | 88 |
| ribosomal protein S10P | 102 | 96 |
| LSU ribosomal protein L3P | 217 | 72 |
| LSU ribosomal protein L4P | 215 | 64 |
| LSU ribosomal protein L23P | 99 | 70 |
| LSU ribosomal protein L2P | 278 | 80 |
| SSU ribosomal protein S19P | 92 | 85 |
| LSU ribosomal protein L22P | 117 | 77 |
| SSU ribosomal protein S3P | 353 | 84 |
| LSU ribosomal protein L16P | 139 | 83 |
| LSU ribosomal protein L29P | 78 | 72 |
| SSU ribosomal protein S17P | 91 | 84 |
| LSU ribosomal protein L14P | 122 | 90 |
| LSU ribosomal protein L24P | 102 | 53 |
| LSU ribosomal protein L5P | 189 | 85 |
| SSU ribosomal protein S14P | 61 | 83 |
| SSU ribosomal protein S8P | 130 | 82 |
| LSU ribosomal protein L6 | 170 | 75 |
| LSU ribosomal protein L18P | 127 | 64 |
| SSU ribosomal protein S5P | 210 | 84 |
| LSU ribosomal protein L15P | 151 | 72 |
| protein translocase subunit secY/sec61 alpha | 438 | 67 |
| adenylate kinase | 186 partial | 62 |
